# Stimulation of Cortico-Subthalamic Projections Amplifies Resting Motor Circuit Activity and Leads to Increased Locomotion in Dopamine-Depleted Mice

**DOI:** 10.3389/fnint.2017.00024

**Published:** 2017-09-28

**Authors:** Teresa H. Sanders

**Affiliations:** Pharmacology Department, Vanderbilt University, Nashville, TN, United States

**Keywords:** STN, deep brain stimulation, hyperdirect pathway, dopamine depletion, 6-OHDA, cortico-subthalamic, optogenetic, Parkinson’s disease

## Abstract

Deep brain stimulation (DBS) of the subthalamic nucleus (STN) improves motor function in patients with Parkinson’s disease (PD). STN-DBS enables similar improved motor function, including increased movement speed (reduced bradykinesia), in the 6-OHDA dopamine-depletion mouse model of PD. Previous analyses of electrophysiological recordings from STN and motor cortex (M1) have explored signaling changes that correspond to PD and amelioration of PD symptoms. The most common results show an increase in beta frequency power during ‘off’ states and a reduction in beta during ‘on’ states. Surprisingly, however, few studies have analyzed whole signal measures of amplitude and coherence during stimulation in freely moving subjects. In previous work by the author, specific transfection of layer five motor cortex projections to the STN revealed an axonal network with collaterals reaching to multiple non-dopaminergic subcortical areas of the brain. The large excitatory shift that stimulation of this axonal network could potentially induce inspired the current study’s hypothesis that amplification of excitatory signaling occurs during stimulation of cortico-subthalamic projections. The results show that, in awake mice, (1) the root-mean-square amplitudes of STN and M1 local field potentials (LFPs) are significantly decreased ipsilateral to chronic unilateral 6-OHDA lesions, (2) stimulation of cortico-subthalamic projections increases the amplitude of M1- and STN-LFPs, and 3) M1-LFP amplitude correlates strongly with locomotion speed in lesioned mice. Together, these findings demonstrate that bradykinesia-reducing stimulation of cortico-subthalamic projections amplifies both cortical and subcortical motor circuit activity in unilaterally dopamine-depleted mice. Most PD treatments are focused on increasing dopamine in the dorsal striatum. However, in this study, stimulation of layer five cortico-subthalamic glutamatergic axons that do not directly project to dopaminergic neurons increased movement and amplified cortico-subthalamic excitatory signaling in dopamine-depleted mice. The correlation between M1-LFP amplitude and locomotion speed observed in these mice points to a role for upregulated hyperdirect pathway excitatory signaling in bradykinesia amelioration. In addition to providing insight into the elusive mechanisms of DBS, these motor circuit amplification relationships suggest that specific manipulation of NMDA, AMPA, and/or metabotropic glutamate receptors in the hyperdirect pathway may be beneficial for upregulating signaling and movement in PD.

## Introduction

The basal ganglia are an ensemble of subcortical nuclei critically involved in controlling action (DeLong, 1990; Hikosaka et al., 2000; Galvan and Wichmann, 2008; Kravitz et al., 2010; Turner and Desmurget, 2010; Mallet et al., 2016). The subthalamic nucleus (STN), the sole glutamatergic nucleus in the basal ganglia, is in a unique and powerful position to influence the processing of motor information. The STN receives glutamatergic inputs from multiple areas of the cortex (Petreanu et al., 2009; Janssen et al., 2016), and is located at the crux of two primary basal ganglia pathway outputs (indirect and hyperdirect, Smith et al., 1998; Nambu et al., 2002).

The three identified basal ganglia pathways, direct (from dopamine receptor D1 expressing medium spiny neurons (MSNs) in the dorsal striatum (dStr)), indirect (from dopamine receptor D2 expressing dStr-MSNs through globus pallidus externa (GPe) and STN) and hyperdirect (cortico-STN), work together to control movement. In go/pause/stop behavioral tasks, the direct and hyperdirect pathways initiate movement in response to a ‘go’ cue. The hyperdirect pathway can then quickly actuate a pause in response to the ‘pause’ signal, while slower processing in the indirect pathway eventually achieves a full stop (Schmidt et al., 2013; Mallet et al., 2016). Excessive spurious synchronization throughout the basal ganglia resulting from dopamine depletion (Hammond et al., 2007) degrades the precise neuronal activity required to implement this normal motor behavior.

**FIGURE 1:**
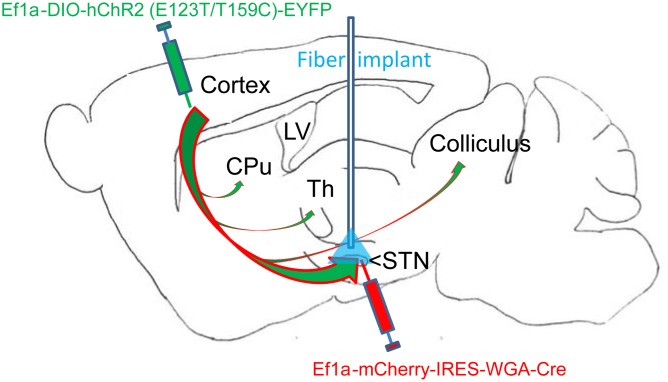
Overview of dual virus injections and optogenetic fiber placement. Virus 1: Adeno-associated virus (AAVs) containing genetic constructs for Cre recombinase (Cre) conjugated to retrograde tracer (WGA) were injected in the subthalamic nucleus (STN). Virus 2: AAVs containing constructs for Cre-dependent ultrafast channelrhodopsin (ChR2) with EYFP fluorophore were injected in motor cortex (M1) layer 5. The two viruses together resulted in specific ChR2 placement in the layer 5 M1-STN projections transfected with retrogradely transported Cre from virus 1. The optogenetic fiber location for STN targeting is labeled in blue. M1-STN terminals and collaterals in striatum (CPu), thalamus (Th), and colliculus identified by EYFP tracing are marked with arrows.

Previous studies demonstrated that projections to the STN from layer 5 (L5) primary motor cortex (M1) neurons extend extensive collaterals in the ipsilateral rodent brain (**Figure 1**; Kita and Kita, 2012; Sanders and Jaeger, 2016), and that the projections undergo remodeling following perturbation of dopaminergic signaling in non-human primates (Mathai et al., 2015). These findings further argue for the relevance of the cortex-to-STN hyperdirect pathway in basal ganglia disorders. However, based on the many conflicting reports in the literature regarding how this pathway is changed in dopamine depleted states and during STN stimulation (Moro et al., 2002; Grill et al., 2004; Gradinaru et al., 2009; Agnesi et al., 2015), additional research is needed to elucidate hyperdirect pathway signaling and associated plasticity both locally and within the context of cortico-basal ganglia networks.

Subthalamic nucleus single cell electrophysiological changes have been found to correlate more strongly with the parkinsonian phenotype than single cell changes recorded in other basal ganglia nuclei (Sanders et al., 2013a; Deffains et al., 2016). This suggests that excitatory (glutamatergic) signaling in the hyperdirect pathway plays a role in the basal ganglia circuit alterations that result in parkinsonian motor dysfunction. Since shared glutamatergic inputs from the cortex directly influence the activity level and synchrony among STN neurons, L5 M1 inputs to the STN are also likely to be important in parkinsonian dysfunction.

The recent finding that M1-STN projection stimulation is sufficient to ameliorate bradykinesia in lesioned mice (Sanders and Jaeger, 2016) provides evidence that M1-STN hyperdirect pathway projections play a primary role in recovery of motor function. The current study uses the dual virus transfection approach employed in the Sanders and Jaeger (2016) study to examine the hypothesis that amplification of excitatory signaling occurs in the hyperdirect pathway during pro-kinetic optogenetic stimulation of cortico-subthalamic projections. The results reveal that M1-STN projection stimulation amplifies M1-STN hyperdirect pathway excitatory signaling as well as movement speed in 6-OHDA unilaterally lesioned mice. Specifically, while M1 and STN LFP amplitudes are reduced in 6-OHDA lesioned mice, stimulation of L5 M1-STN projections increases M1 and STN LFP amplitude and produces speed of movement that is correlated with M1-LFP amplitude in these mice. Since STN-DBS is the most effective and widely prescribed DBS treatment for PD (Benabid et al., 2009; Odekerken et al., 2016), these findings regarding hyperdirect pathway signaling changes in parkinsonism and during stimulation are valuable for advancing treatment for PD patients. In particular, the findings suggest measures that can be used to improve STN-DBS (Wingeier et al., 2006) and identify potential hyperdirect pathway therapeutic targets.

## Materials and Methods

### Experimental Procedures

This study was carried out in accordance with the National Research Council Guide for the Care and Use of Laboratory Animals, the PHS Policy on the Humane Care and Use of Laboratory Animals, and the American Physiological Society’s Guiding Principles for the Care and Use of Vertebrate Animals in Research and Training. The protocol was approved by the Emory Animal Care and Use Committee.

### Animals

Male C57BL/6J mice (JAX Labs) were housed with ad libitum access to chow and water in environmentally controlled conditions. Mice were placed on a 12 h reversed light/dark cycle (lights on at 7 pm).

### Mouse Surgeries

Surgeries were performed as previously described in Sanders and Jaeger (2016). Briefly, to model severe dopamine (DA) depletion, 6-OHDA (Sigma) was injected in the medial forebrain bundle (-1.2 AP, -1.2 ML, -4.75 DV, coordinates from Paxinos and Franklin, 2004) of mice at P24–28 to unilaterally lesion DA neurons. The extent of the lesion was measured with limb use asymmetry and bradykinesia and verified post-mortem with tyrosine hydroxylase (TH) staining (Cenci and Lundblad, 2007). In the same surgery, adeno-associated virus (AAVs; Deisseroth lab) carrying ultrafast (second generation) Cre-inducible channelrhodopsin (ChR2)-EYFP constructs (Ef1a-DIO-hChR2(E123T/T159C) – EYFP) (Berndt et al., 2007; Jackman et al., 2014) were injected into M1 while AAVs carrying a retrograde tracer – Cre construct (Ef1a-mCherry-IRES-WGA-Cre) were injected in STN. Opsins were expressed at the intersection of the retrogradely placed Cre and the Cre-dependent ChR2 (i.e., M1- STN projections). An appropriate titer (2 × 10^12^ genomic copies/ml), volume (0.36 μl), and coordinates (STN: AP -1.76 mm AP, -1.56 mm ML, -4.2 mm DV and M1: 2.0 mm AP, -1.56 mm ML, and -1.0 mm DV from bregma) was used to ensure transfection of a consistent, large population of the desired neurons with minimal off-target spread of the virus. All injections and subsequent animal housing were carried out with appropriate BSL handling procedures and were approved by Emory University IACUC.

The dual virus transfection approach enabled selective optogenetic activation of glutamatergic projections (Sanders and Jaeger, 2016). A second surgery was performed 3-weeks after the virus injection surgery. 50 μm Pt/Ir recording electrodes were placed in M1 and STN. A ground wire was attached to a skull screw above the cerebellum. All wires were connected to an electronic interface board and affixed to the skull using dental cement. Additionally, dental cement was used to attach a stereotaxically implanted 200 μm optical fiber stub with tip located approximately 50–150 μm above the transfection site either in layer 5 M1 (*n* = 1 mouse) or STN (*n* = 8 mice). The fiber stub was attached to the STN (or M1) recording electrode before implantation to ensure stimulation and recording occurred in close proximity. The fiber tip was placed slightly below the electrode tip to avoid light artifacts. Recording was performed 4–6 weeks postoperatively. Note that the electrophysiological and locomotion speed data from the cortically stimulated mouse did not differ statistically from the data recorded in the STN-stimulated mice, therefore the data from the two stimulation sites were pooled for further analysis.

### Histology

After all experiments were completed, mice were perfused transcardially with phosphate buffered saline followed by a 4% formaldehyde solution. Brains were removed and then sectioned with a freezing microtome (50–60 μm sections). Sections were mounted on slides for further processing.

**FIGURE 2:**
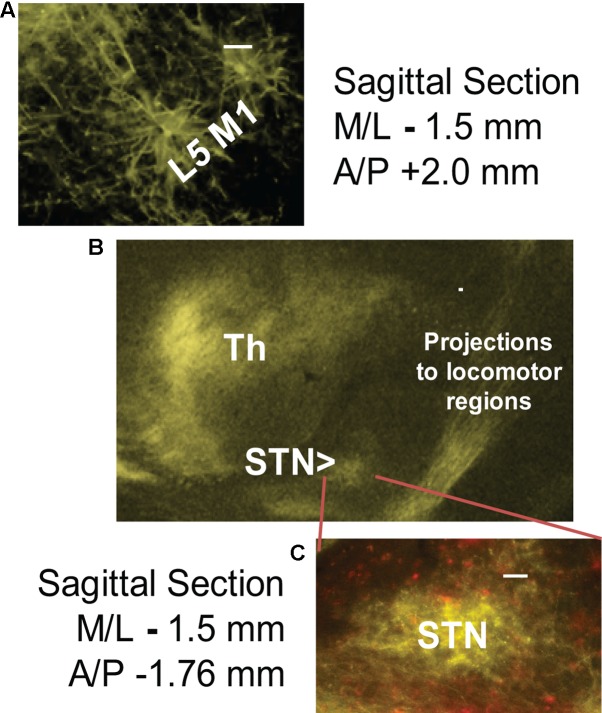
Successful cortico-subthalamic (M1-STN) projection transfection was confirmed by the presence of EYFP (yellow) in **(A)** layer 5 M1 pyramidal cells, **(B)** M1 descending projections and collaterals, and **(C)** M1-STN terminals. mCherry (red) staining indicates WGA/Cre construct injection site. The middle panel **(B)** shows the opsin transfection pattern in the region around the STN. Scale bars = 100 μm.

6-OHDA lesioning was confirmed through TH staining quantification. Successful transfection of M1-STN projections and neuron morphology was evaluated by mapping of EYFP on consecutive slides through all structures from cortex to collaterals and from cortex to STN (**Figures 1**, **2**).

### Behavioral and Electrophysiological Recording

Cameras and head-mounted accelerometers were used to assess open field movement concurrent with electrophysiology (Intan Technologies). To facilitate free movement, electronic cables and fibers connected to the mouse were passed through a power-assisted commutator (Doric) before being connected to data acquisition hardware (Spike2; Cambridge Electronic Design) and laser light source. Local field potentials (LFPs) were recorded from electrodes.

### Behavioral Analysis

Mice were recorded in an open-field arena for 5 min with no stimulation, followed by 3 periods of 5-min stimulation with each period using one of three frequencies [30 Hz (4 ms pulsewidth), 100 Hz (4 or 5 ms pulsewidth), or 130 Hz (4 ms pulsewidth)]. Frequencies were randomized and rest periods provided between each stimulation period to avoid effects due to stimulation order. Experiments for which locomotion occurred were analyzed for movement speed (bradykinesia) and fraction of total seconds with movement using MATLAB. The fraction of seconds with movement measure was calculated by dividing the total number of one second intervals where movement exceeded 4 mm/s by the total number of seconds in the trial. During 30 Hz stimulation trials, locomotion (Sanders and Jaeger, 2016) and electrophysiology were not significantly changed from the unstimulated trials. Therefore, only data from the unstimulated and high-frequency (100 Hz and 130 Hz) optogenetically stimulated trials were included in the current study.

### Analysis of Electrophysiological Data

As calculated in the preliminary results and previous publication, measures found to be important in humans and animal models (Lopez-Azcarate et al., 2010; Sanders et al., 2013b; Sanders, 2016; Shreve et al., 2016) were used to estimate signaling within and between M1 and STN. Measures such as phase-amplitude-coupling and band power assessed in the previous study of cortico-subthalamic stimulation (Sanders and Jaeger, 2016) were not included here, unless their inclusion shed light on the electrophysiological measures examined in the current study. Any such inclusions are indicated in the text.

In order to reduce the potential for movement artifacts and ensure that all electrophysiology was assessed in a common state, only LFPs captured during wakeful, non-moving periods were analyzed. All such segments that met the quality criteria of noise standard deviation < 200 μs and had no visible frequency or other artifacts were downsampled from the original 20 kHz recording rate to 1000 Hz, then band-pass filtered between 3 and 500 Hz before calculating the following measures. The root-mean-square (RMS) amplitude was calculated for each segment using the standard definition,

$$V_{RMS}=\sqrt{\frac{1}{n}\Sigma_{i=1}^{n}{LFP}_{i}^{2}.}$$

Correlation coefficients and correlation statistical significance were determined using the MATLAB corrcoef function. Coherence was calculated using the MATLAB mscohere function.

### Experimental Design and Statistical Analysis

The effects of optogenetic cortico-subthalamic stimulation on bradykinesia and STN and M1 population signal amplitudes were examined in C57BL/6J mice 2–4 months post lesion. The effect size chosen was 0.58 since this represented a 20% change in the speed per trial based on the mean and standard deviation of the 6-OHDA mice observed in previous analysis (Sanders and Jaeger, 2016). To achieve this effect at a power of 0.8 and significance level of 0.05 with a paired *t*-test, a minimum of *n* = 26 paired data samples were required (“n” determined using R power analysis package, “pwr”). For unpaired measures, a minimum of *n* = 48 samples from each data group were necessary to achieve a power of 0.8. Statistical analyses for unpaired measures were performed only if a minimum of 48 samples from each group were collected.

Nine mice were analyzed to confirm bradykinesia in lesioned mice and amelioration with stimulation (Sanders and Jaeger, 2016). Five of these mice were found to have complete, low noise STN- and M1-LFP recordings (three 6-OHDA lesioned and two controls). M1-LFP power and associated speed of movement were evaluated for all such trials involving these mice in the current study (19 trials; a total of 4680 s of data analyzed).

Note that electrophysiological measures were assessed for non-moving segments in which the animal was awake in order to avoid including potential movement artifacts and slow wave sleep signals in the analysis. Increased locomotion (i.e., reduced bradykinesia) was assessed with the Kolmogorov–Smirnov test since calculation of cumulative distribution statistics was possible due to the large quantity of accurate velocities (1s samples) obtained from the automated movement analysis scripts. Measures taken from the same subjects before and after manipulation were assessed using the paired *t*-test (LFP RMS amplitudes). For non-paired measures that were pooled across mice, Wilcoxon ranksum tests were used.

The primary results that speed and LFP amplitudes were increased in stimulated lesioned mice were found to be significant using the methodology described above. For comparison purposes, unlesioned control data with the same opsin transfection and implants were also evaluated.

## Results

Optical fibers implanted near M1-STN projections in the left hemisphere successfully stimulated opsins specifically transfected in these projections. Recording electrodes placed in M1 and STN successfully measured neural activity with adequate signal-to-noise ratio in all mice included in the study. The RMS amplitude of the population signals (M1- and STN-LFPs) during non-moving (resting) segments and the speed of movement were reduced in awake DA-depleted (6-OHDA lesioned) mice as compared to awake controls. During high frequency optogenetic stimulation (HFOS, 100 Hz or 130 Hz), the resting M1- and STN-LFP RMS amplitudes and movement speed in these lesioned mice were both increased in lesioned mice.

### Optogenetic Stimulation (100 or 130 Hz) Modulated LFPs

**FIGURE 3:**
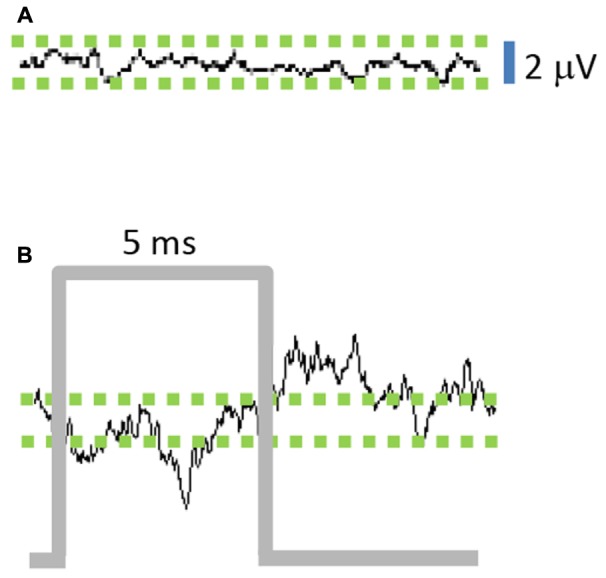
Stimulation of M1-STN projections modulates local field potential (LFP) amplitude. **(A)** Average of 10 ms epochs of STN-LFPs in absence of stimulation results in small residual signal due to random variations between epochs, while **(B)** Stimulus-triggered average of 10 ms epochs reveals amplification effect of stimulation on STN-LFPs. (*n* = 1000 epochs for **A,B**).

The average of STN-LFPs aligned to the start of each stimulus pulse (stimulus-triggered average, 100 Hz stimulation frequency) shows a negative deflection during the stimulus pulse (**Figure 3B**), followed by a positive deflection in the interval after the pulse. The peak-to-peak stimulus-triggered average amplitude of the stimulated STN-LFP signal increased by a factor of 4.25 compared to the unstimulated signal (**Figure 3A**), indicating that the optogenetic stimulus modulated the LFP signal (*n* = 1000, *p* = 0.003).

### Cortico-Subthalamic Stimulation Increased Resting M1- and STN-LFP Amplitudes in Lesioned Mice

**FIGURE 4:**
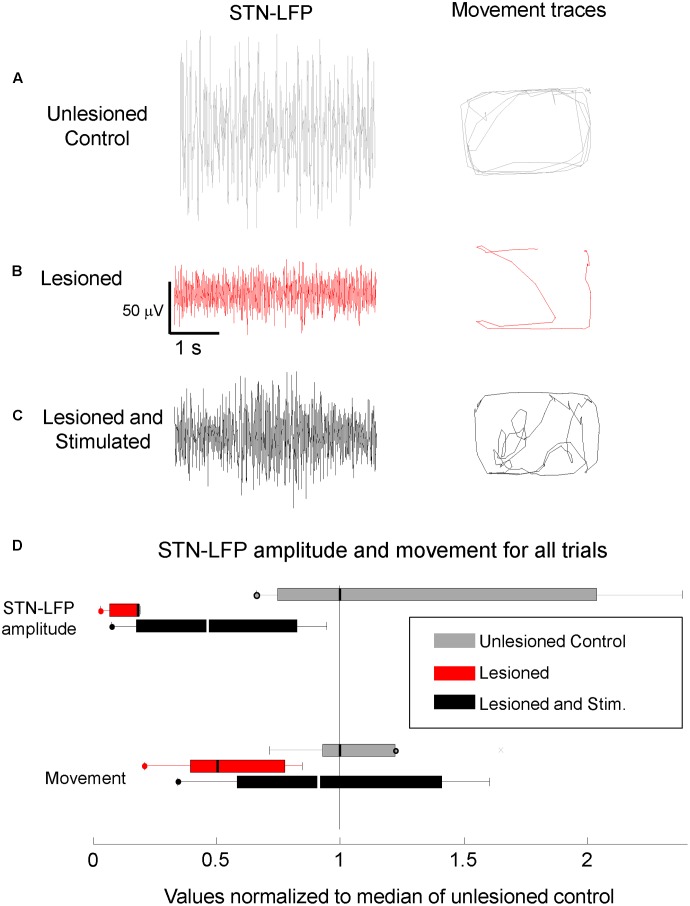
Reduced STN-LFP amplitude and movement in lesioned mice were partially restored by M1-STN projection stimulation. Representative sample STN-LFP and movement for **(A)** unlesioned control, **(B)** 6-OHDA lesioned, and **(C)** M1-STN stimulated mice with 6-OHDA lesions, **(D)** Box-and-whisker plots summarizing STN-LFP amplitudes and movement for control, lesioned, and stimulated mice relative to control values. Circles at the end of whiskers indicate there are no data points outside the whisker. Note that the representative locomotion samples illustrate the typical amount of locomotion in each condition, not the pattern of movement. No consistent differences in the amount of time spent in the center versus the edges of the open field were observed between the stimulated lesioned mice and controls.

Local field potentials in mice with 6-OHDA lesions were recorded in the left hemisphere before, during, and after stimulation. Resting STN-LFP RMS amplitudes were reduced in lesioned mice as compared to controls and partially restored by M1-STN projection stimulation (**Figure 4**, *n* = 5 mice, paired *t*-test, *p* = 0.036). Similarly, resting M1-LFP RMS amplitudes were reduced in lesioned mice as compared to controls and partially restored by M1-STN projection stimulation. The ratio between the average M1-LFP RMS amplitude in stimulated versus unstimulated trials was 3.5648 (three mice × two conditions (unstimulated, stimulated), *n* = 612 s, paired *t*-test, *p* = 0.0018).

### Cortico-Subthalamic Stimulation Increased Movement in Lesioned Mice

**FIGURE 5:**
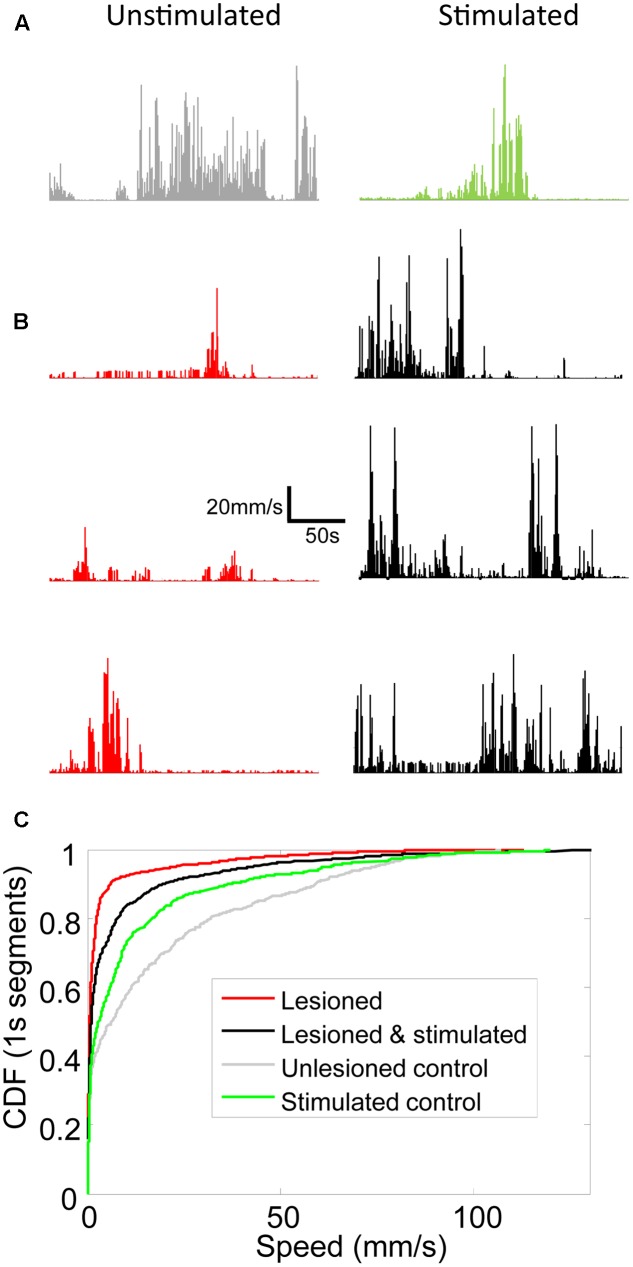
Locomotion speed increased in lesioned mice receiving stimulation. Line graphs showing locomotion speed for each 1s of representative 5 min trials for **(A)** unlesioned control and **(B)** 3 individual lesioned mice. Left column shows unstimulated trials. Right column shows optically stimulated (100 Hz or 130 Hz) trials. **(C)** Cumulative distribution functions (CDFs) of locomotion speed for all trials in unlesioned control and lesioned mice with and without stimulation. Rightward shift of curve indicates more high speed 1s segments.

**FIGURE 6:**
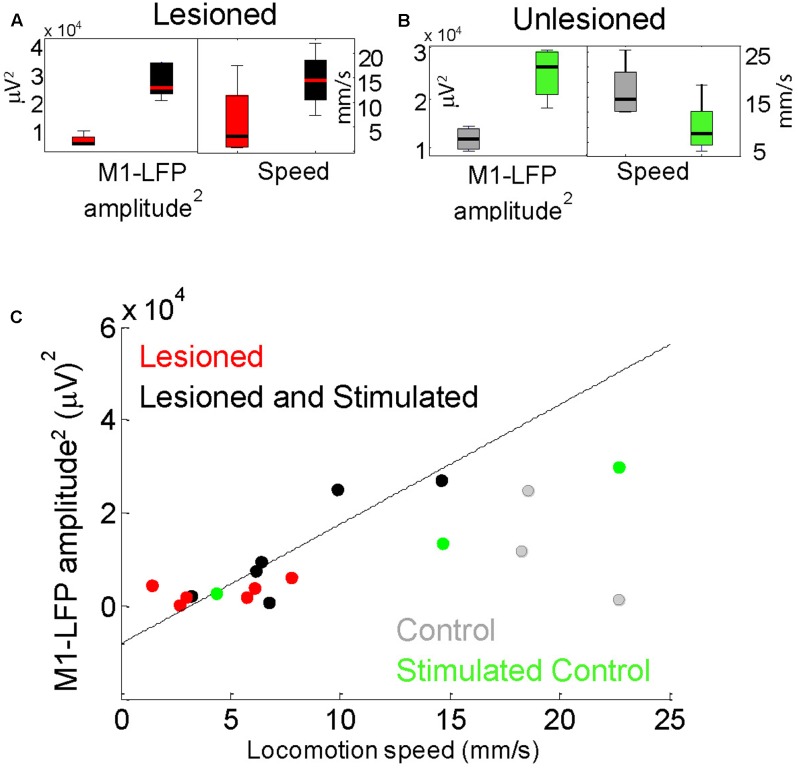
Locomotion speed correlated with M1-LFP signal amplitude in lesioned mice. **(A)** M1-LFP root-mean-square (RMS) amplitude’ and locomotion speed were increased during stimulation in lesioned mice (boxplots reflect data from each minute in a representative 5-min trial). **(B)** M1-LFP RMS amplitude was increased and locomotion speed was decreased in unlesioned control. **(C)** M1-LFP RMS amplitude vs. mean locomotion speed for all trials. Each point represents a 5-min trial. Dashed line indicates a linear fit to the points for the lesioned mice.

Movement and locomotion speed in lesioned mice were measured before, during, and after stimulation. As reported in Sanders and Jaeger (2016), movement was reduced in lesioned mice as compared to controls, and was partially restored by M1-STN projection stimulation (**Figure 4**). Locomotion speed was also reduced in lesioned animals as compared to controls. Stimulation increased locomotion speed in lesioned mice but reduced control locomotion speed (**Figures 5**, **6A,B**). (*n* = 9120s, Kolmogorov–Smirnov test; lesioned vs. control *p* = 1.6977e-063, lesioned with stimulation vs. lesioned *p* = 1.4477e-021, unlesioned with stimulation vs. unlesioned *p* = 2.4722e-006).

### M1-LFP Amplitude and Speed of Movement in Lesioned Mice Were Correlated

For each 5-min trial, STN- and M1-LFP RMS amplitudes during non-moving segments were compared with the average locomotion speed during that trial. As reported in Sections “Cortico-Subthalamic Stimulation Increased Resting M1- and STN-LFP Amplitudes in Lesioned Mice” and “Cortico-Subthalamic Stimulation Increased Movement in Lesioned Mice,” both STN-LFP RMS amplitudes and locomotion speeds were significantly increased in lesioned mice during stimulation. However, STN-LFP amplitudes and locomotion speeds did not correlate significantly. In contrast, M1-LFP amplitudes increased in a correlated manner with increasing locomotion speed in lesioned mice (**Figures 6A,C**, *n* = 12 trials, correlation coefficient *r* = 0.8405, *p* = 0.0006). In the control, M1-LFP amplitudes increased during stimulation (**Figure 6B**) but did not correlate positively or negatively with locomotion (**Figure 6C**, *n* = 6 trials, *r* = 0.4433, *p* = 0.3787). These results are consistent with the observed improved contralateral movement in lesioned mice during stimulation. The latter result suggests that unilaterally increasing excitatory signaling (and possibly unilateral movement) may impede overall locomotion in controls (see section “Discussion”). Note that when only stimulated lesioned mice were considered, the number of trials was the same as for the control, however, the correlation between M1-LFP amplitude and locomotion speed remained significant (*r* = 0.8858, *p* = 0.0188).

### Differences in Coherence Changes between Lesioned and Unlesioned Mice

**FIGURE 7:**
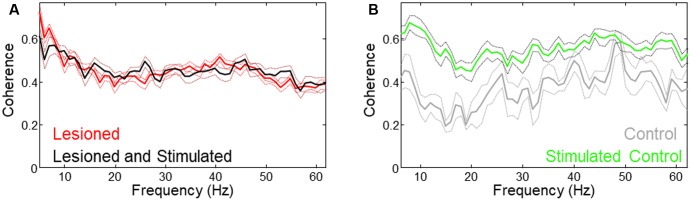
Different coherence changes occurred with stimulation in lesioned vs. unlesioned mice. **(A)** Low frequency coherence was reduced in lesioned mice during stimulation. **(B)** Coherence was increased in unlesioned control during stimulation.

Mean coherence was increased in lesioned mice as compared to controls. As reported in the previous study (Sanders and Jaeger, 2016), theta coherence decreased during stimulation in lesioned mice. Evaluation of whole-signal coherence in this study showed no mean increase in stimulated lesioned mice, but revealed a significant increase in the stimulated control (**Figure 7**, three mice × two conditions (unstimulated, stimulated), *n* = 612 s, *p* = 0.015).

## Discussion

This study found that STN-LFPs are amplified in M1-STN stimulated mice (**Figures 3**, **4**), and that resting M1-LFP RMS amplitude correlates with mouse locomotion speed in awake DA-depleted mice (**Figure 6**; note that hereafter “RMS amplitude” is referred to as “amplitude”). The results further show that M1- and STN-LFP amplitude and mouse movement are reduced in lesioned mice compared to unlesioned mice, and that M1- and STN-LFP amplitude and mouse movement are increased by M1-STN projection stimulation (**Figures 4–6**). These results suggest that upregulated hyperdirect pathway excitatory signaling is closely related to locomotion speed recovery in DA-depleted mice. Similar upregulated hyperdirect pathway signaling may be at least partially responsible for the amelioration of bradykinesia experienced by PD patients receiving STN-DBS treatments. While this is not consistent with studies that propose an STN lesioning effect occurs in therapeutic DBS (Filali et al., 2004; Grill et al., 2004), it is supported by electrophysiological studies showing that STN activity correlates more strongly with basal ganglia downstream activity than striatal activity (Deffains et al., 2016) and that STN activity is increased during DBS (Garcia et al., 2005).

Although in some cases increased LFP amplitude is associated with greater synchronization between neurons, the results in this study suggest that synchronization (as measured by coherence) during stimulation is not related to the increased power. This is supported by the result that coherence is increased only for the control despite increased M1- and STN-LFP amplitude in both lesioned and control animals (**Figure 7**).

Many other sequelae of stimulation such as changes in beta band power, coefficient of variation, and entrainment of neural activity at particular frequencies have been studied at length, but the seemingly obvious measures of broad spectrum LFP amplitude examined in this study appears to have been largely overlooked. The study’s results regarding significant changes in signal amplitude between unlesioned, lesioned, and stimulated mice, and the strong correlation between stimulated locomotion speed and M1 signal amplitude in lesioned mice suggest the measure may be a useful cortico-basal ganglia circuit analysis tool and biomarker for both dopamine depletion and effective STN stimulation.

### Neural Mechanisms

Glutamate receptors in the cortex and STN exhibit alterations in DA-depleted conditions (Chu et al., 2015), including increased excitability and response to bath application of glutamate and GABA as compared to controls. Shen and Johnson (2005), also report a shift in ionotropic glutamate receptors expression away from NMDA receptors and a concomitant increase in the AMDAR/NMDAR whole cell current in the STN of lesioned mice. The increased STN current and excitability observed at the single cell level in 6-OHDA lesioned mice and the increased synchronization reported in parkinsonian circuit activity (Hammond et al., 2007) would seem to be at odds with the reduced STN-LFP amplitude observed in this study. However, it is important to note that increased cellular excitability and bursting have been shown to shift NMDA receptor expression from synaptic to extra-synaptic locations (Papouin et al., 2012) often due to pathological release of glutamate from astrocytes that occurs as a by-product of neurodegeneration (Parsons and Raymond, 2014). Increased extra-synaptic NMDA receptor expression has, in turn, been observed to promote synchrony, long-term-depression (LTD, Lüscher and Malenka, 2012), and cell death (Paoletti et al., 2013; Yashiro and Philpot, 2008). Thus, one explanation for the decreased LFP amplitude in the chronic 6-OHDA lesioned mice in our study is that such a shift toward extra-synaptic NMDA receptor expression has occurred, resulting in reduced excitatory population signal amplitude (along with chronic bursting and synchrony).

In contrast, stimulation applied near motor cortical terminals at the STN (e.g., optogenetically as in this study, or as in electrical STN-DBS) may lead to increased synaptic activity that propagates antidromically and orthodromically, increasing the cortico-subthalamic population activity (and LFP amplitude) relative to the extra-synaptic activity that predominates in the unstimulated lesioned hyperdirect pathway. Over time, such stimulation would likely shift the balance of NMDA receptor expression back toward the synapses being stimulated, and away from extra-synaptic locations.

### Circuit Mechanisms

Circuit-level alterations within the hyperdirect pathway during lesioning and stimulation are supported by the data showing that the M1 and STN signal strength (**Figures 4**,**6C**), synchronization (**Figure 7**), and STN phase-amplitude coupling (PAC, Sanders, 2016; Sanders and Jaeger, 2016) all significantly change following a chronic 6-OHDA lesion, and are restored to more normal levels during M1-STN projection stimulation. It is likely that M1-STN signaling alterations are similarly propagated throughout the basal ganglia in PD and during STN-DBS.

It is important to note that, as reported in the previous related study (Sanders and Jaeger, 2016), differences exist in the STN-LFP and the M1-LFP changes during lesioning and stimulation. In the current study, M1-LFP amplitude correlated with locomotion speed, while STN-LFP amplitude did not. Considering the results from the two studies jointly suggests that the main effect of stimulation at the STN may be to reduce spurious bursting (as measured by PAC), thereby enabling more normal cortico-basal ganglia circuit dynamics and allowing M1 to produce excitatory signaling that directly increases locomotion.

Since M1-STN stimulation immediately impacted movement in our study, the effects could not be entirely due to plasticity in the circuit. As described in the *Neural mechanisms* section above, the location of stimulation near the synapses between the layer 5 motor cortex terminals and the STN could immediately bias M1-STN signaling toward synaptic channels (and away from extra-synaptic inputs), leading to amplification of orthodromic and antidromic signaling. Other factors such as increased nitric oxide signaling and vasodilation, along with upregulated neural clearance may have played a role in the short term increases in movement and signaling.

The lack of significant increased movement in the control animal argues against an overall increased motivation (reward) effect due to cortico-subthalamic stimulation. Instead the stimulation may have directly increased motor signaling thereby helping rebalance the weakened contralateral motor abilities in the lesioned mice while unbalancing the unlesioned (already balanced) control. This unbalancing effect could be related to the strongly increased M1-STN coherence observed in the unlesioned control that was not observed in the lesioned mice (**Figure 7**).

The increased signal amplitude in cortico-subthalamic projections likely leads to upregulation of movement circuitry via the multiple excitatory projections in the stimulated hemisphere (e.g., mesencephalic locomotor areas, as reported in Sanders and Jaeger, 2016). However, unchecked upregulated STN signaling would be expected to reduce movement by increasing inhibition from the entopeduncular nucleus/substantia nigra pars reticulata (EP/SNr) to the thalamus (thereby reducing thalamic outputs to cortex). It is important to note that due to network effects, STN outputs are tempered during cortico-subthalamic stimulation (and STN-DBS), since STN outputs to basal ganglia output nuclei (EP/SNr) may be modified by stimulation-driven STN orthodromic activation of sub-populations of inhibitory GPe cells (Mastro et al., 2017) which project back to STN (Chu et al., 2015). Antidromic activation (Li et al., 2007) of cortical circuitry may impact the effects of STN outputs through circuit effects as well.

### Experimental Considerations

Although histological evidence indicates that the desired projections were transfected, it is possible that not all the projections identified represent monosynaptic connections. However, since the stimulation in this study was applied locally at cortico-subthalamic terminals, it is likely that the wider spread projections identified histologically are not directly activated, but rather indirectly activated through stimulation of opsins in the more proximal areas.

Movement artifacts can be a potential source of crosstalk in LFP recordings. For this reason, the electrophysiological analysis in the current study was performed on the non-moving epochs within each trial. Fortunately, the intermittent movements of the mice throughout the 5 min trials (**Figure 5**) provided sufficient data to enable analysis at the desired statistical power and significance as discussed in the methods.

The small number of non-lesioned controls in the study (*n* = 2 unstimulated; *n* = 1 stimulated) limits the conclusions that can be drawn regarding control behavior and relative comparisons with the lesioned mice. However, the repeatable results observed over multiple days of trials in the control, and the clear difference between the control and lesioned trials, prompted the inclusion of the data in this article.

### Implications for Treatment

Stimulation of cortico-subthalamic projections in the ipsilateral brain of 6-OHDA lesioned mice increases movement speed and hyperdirect pathway signaling. Although there may be differences in the response of cortico-subthalamic circuits in electrical DBS and the optical stimulation used in this study, the finding that hyperdirect pathway stimulation specifically addresses bradykinesia provides potentially useful information for PD treatment selection. Additionally, the correlation between movement speed and M1-LFP amplitude suggests a straightforward relationship that may be useful for adjustment of DBS stimulation settings.

Finally, the electrophysiological changes associated with the increased movement indicate that stimulation amplifies excitatory signaling toward the amplitude seen in unlesioned controls. These motor circuit amplifications suggest that manipulation of NMDA, AMPA, and/or metabotropic glutamate receptors in the hyperdirect pathway might be beneficial for upregulating signaling and movement in parkinsonian patients.

## Author Contributions

TS designed the work, performed the experiments, and collected, analyzed, and interpreted the data. TS also drafted the manuscript and approved the final version.

## Conflict of Interest Statement

The author declares that the research was conducted in the absence of any commercial or financial relationships that could be construed as a potential conflict of interest.
